# Non-Invasive Optical Coherence Tomography Data-Based Quantitative Algorithm for the Assessment of Residual Adhesive on Bracket-Removed Dental Surface

**DOI:** 10.3390/s21144670

**Published:** 2021-07-07

**Authors:** Yoonseok Kim, Gu-In Jung, Deokmin Jeon, Ruchire Eranga Wijesinghe, Daewoon Seong, Jaeyul Lee, Woo Jong Do, Sung-Min Kwon, Jong Hoon Lee, Jun Ho Hwang, Hyun Deok Kim, Kyu-Bok Lee, Mansik Jeon, Jeehyun Kim

**Affiliations:** 1 School of Electronic and Electrical Engineering, College of IT Engineering, Kyungpook National University, Daegu 41566, Korea; otter0618@knu.ac.kr (Y.K.); deokmin.jeon@gmail.com (D.J.); smc7095@knu.ac.kr (D.S.); jaeyul@knu.ac.kr (J.L.); hdkim@knu.ac.kr (H.D.K.); jeehk@knu.ac.kr (J.K.); 2 Institute of Advanced Convergence Technology, Kyungpook National University, Daegu 41061, Korea; guin83@knu.ac.kr (G.-I.J.); loseo@knu.ac.kr (W.J.D.); sungmin@iact.or.kr (S.-M.K.); laser@iact.or.kr (J.H.L.); hjh@iact.or.kr (J.H.H.); 3 Department of Materials and Mechanical Technology, Faculty of Technology, University of Sri Jayewardenepura, Pitipana, Homagama 10200, Sri Lanka; erangawijesinghe@sjp.ac.lk; 4 Advanced Dental Device Development Institute, Kyungpook National University, Daegu 41940, Korea; kblee@knu.ac.kr; 5 Department of Prosthodontics, School of Dentistry, Kyungpook National University, Daegu 41940, Korea

**Keywords:** dental optical coherence tomography, orthodontic imaging, dental adhesive, residual adhesive, bracket removal inspection

## Abstract

The aim of this study was to quantitatively assess the residual adhesive on orthodontic ceramic bracket-removed dental surface. In orthodontic process, ceramic bracket was repeated debonding physically, then the adhesive remained on the dental surface. The residual adhesive caused a lack of adhesive strength between dental and ceramic bracket. Since commonly used adhesive in orthodontics is translucent, residual adhesive is hard to be detected with conventional microscopes. Therefore, 1310 nm center wavelength swept-source OCT system based on laboratory customized image processing algorithm was used for the precise detection of residual adhesive on tooth surface. The algorithm separates residual adhesive from dental surface by comparing the height of adjacent B-scan images, while providing color-scaled images emphasizing the thickness information of residual adhesive. Finally, the acquired results were compared with microscopic and adhesive remnant index scoring gold standards, while the comparison confirmed the potential merits and the improvements of the proposed method over gold standards.

## 1. Introduction

Recently, orthodontics using translucent adhesives and translucent orthodontic brackets have gained popularity. However, frequent replacement of orthodontic brackets can easily cause damage to the enamel surface [1,2,3]. Adhesives and resins are simultaneously used in orthodontics as the robust bonding component of orthodontic brackets to the tooth surface. In particular, the process of attaching and removing the orthodontic prosthesis to the tooth surface is repeated several times in orthodontics [4]. The orthodontic prosthesis is bound to the dental surface using an adhesive, and then physically removed using pliers. The orthodontic process requires the bracket to be reattached to the tooth surface several times. For a perfect fixation of the bracket, residual adhesive has to be entirely removed, since it reduces the bonding strength of the new bracket with the tooth surface. However, a particular amount of the adhesive remains on the tooth surface and degrades the bond between bracket and tooth surface. Thus, in current practice, the amount of residual adhesive is examined visually or physically, and a mechanical drill is utilized to obtain a clear surface. During this mechanical process, the applied extra force simply removes not only adhesive, but also damages the enamel surface leading to side effects such as tooth erosion, coloring, and enamel surface damages [5,6,7,8,9]. Therefore, pre-identification of the approximate amount of residual adhesive can significantly barricade aforementioned damages and minimize unnecessary extra force.

The gold standard of residual adhesive measurement has always been subjective to orthodontists and dentists [10]. The adhesive remnant index (ARI) score is the existing subjective scoring method to evaluate residual adhesives identified with the naked eye or magnification microscope. This unit is called “0” when there is no residual adhesive on the tooth surface, “1” when the residual adhesive is below 50% on the tooth surface, “2” when it is 50% or more, and “3” when it is almost unremoved [11]. Due to subjectivity, invasiveness, and lack of precision in the readings, non-invasive and highly accurate residual adhesive measurement methods have been the focus of enormous interest during the past few decades in the field of orthodontics [12,13].

To evaluate residual adhesive measurements non-invasively, an optical magnification microscope has been the tool most frequently applied [14,15]. However, the optical magnification microscope has limitations when it comes to obtaining boundary information, an essential requirement to analyze the exact edges of residual adhesive. In addition, micro-computed tomography (micro-CT) and cone-beam computed tomography (CBCT) have been widely used as imaging tools for dental applications. Additionally, micro-CT and CBCT are utilized in dentistry and orthodontics applications for the apprehension of structure [16,17,18,19,20]. However, these two imaging tools have several issues of limitation and liability. Computed tomography (CT) technology has a natural liability of exposure to ionizing radiation. Moreover, CT is an expensive imaging tool for patients, which has low resolution, as well as a being a complex time-consuming operating method [21]. To resolve these drawbacks, a high-resolution medical imaging device called optical coherence tomography (OCT) [22,23,24,25] has gained interest in numerous dental and orthodontic applications [26,27,28]. OCT has been extensively employed to examine dental materials at various dental applications. OCT has been widely applied to examine gingival sulcus, dental caries, periodontal tissues, and quantitative gaps between teeth and crown, among others, due to its high-resolution and non-invasiveness [29,30,31,32,33]. Adhesive thickness and the gap between tooth surface and the adhesive were examined using OCT at multiple attempts [34].

In this study, we have used the non-invasive properties of OCT to qualitatively and quantitatively assess residual adhesive on the tooth surface using a laboratory customized image processing algorithm. This algorithm quantifies the residual adhesive on the surface of the teeth to deliver area and thickness information. The fine height difference between teeth and residual adhesives was precisely distinguished using OCT due to its high depth resolution. It also provided visual information about the thickness of the particular region of residual adhesive. This algorithm illustrates thickness information intuitively by color scaling. In addition, images can be obtained by separating only the remaining adhesives present on the surface of the tooth sample using the proposed residual adhesive detection algorithm. Although evaluations of the residual adhesive on the dental surface using OCT were reported, quantitative confirmations obtained for residual adhesive have not been described before.

## 2. Materials and Methods

### 2.1. Sample Preparation

**Figure 1:**
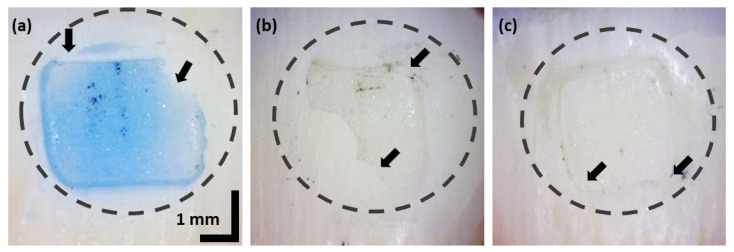
Optical microscope images of samples: (**a**) Sample A with blue color adhesive; (**b**,**c**) Sample B, sample C with transparent adhesive. Black color solid arrows indicate the residual boundaries.

A total of three bovine teeth specimens were used in the study; these teeth were pretreated for the study. To prevent decomposition, all the nerves were successfully removed after tooth extraction. To prevent infection, specimens were stored in a 0.5% chloramine T solution in distilled water at 4 °C for 7 days. All ex vivo tooth specimens were pumiced with a polishing paste for 10 s, cleaned using water for 20 s, surfaces were treated with pumice stone and a rubber cup for 10 s, then washed and dried. Next, the specimens were etched with 37% phosphoric acid gel (CharmEtch^®^-37HV, Dentkist, Inc., Gunpo-si, Gyeonggi-do, Korea) for 20 s, rinsed, dried for 15 s, and adhesive (TransbondTM XT, 3M Unitek, Monrovia, CA, USA) was uniformly applied on each tooth surface. Finally, clarity ceramic brackets (CLARITYTM MBTTM Rx, 3M Unitek, Monrovia, CA, USA) were bound on the surface of each tooth specimen. Prepared samples with adhesive were UV light-cured for 20 s. After the bonding procedures, the samples were stored in water at 37 °C for 24 h to ensure complete polymerization. Adhesive attached to three specimens were used, where two specimens were with blue color adhesive and the remaining one with translucent adhesive. Although both translucent and colored adhesives have been used in orthodontics, the residual of colored adhesives can be clearly identified using conventional microscopic methods. To assess the feasible investigation of both adhesive types using OCT, both translucent and colored adhesives were utilized for the experiment. As shown in Figure 1, blue adhesive was used on sample A, and transparent adhesive was used on samples B and sample C. The adhesive on tooth specimens was removed mechanically. However, confirming the partial or complete removal of adhesive is a challenging task using conventional visual inspections. Thus, the specimens have to be examined using an optical microscope to confirm the status of residual adhesive. The circular dashed regions indicate the residual adhesive regions, and the solid arrows indicate the points of ambiguous boundaries of adhesive, which were examined using a high-resolution optical imaging method for increased precision. The bovine teeth specimens had a curved shape into a protruding center. The curved shape of the tooth surface varies slightly from sample to sample, but most of them are higher in the center. Due to the different shapes of most samples, a laboratory customized image processing algorithm (coded in MATLAB, Natick, MA, USA) was developed to quantitatively analyze the area and thickness of the residual adhesive on the tooth surface.

### 2.2. OCT System Configuration

**Figure 2:**
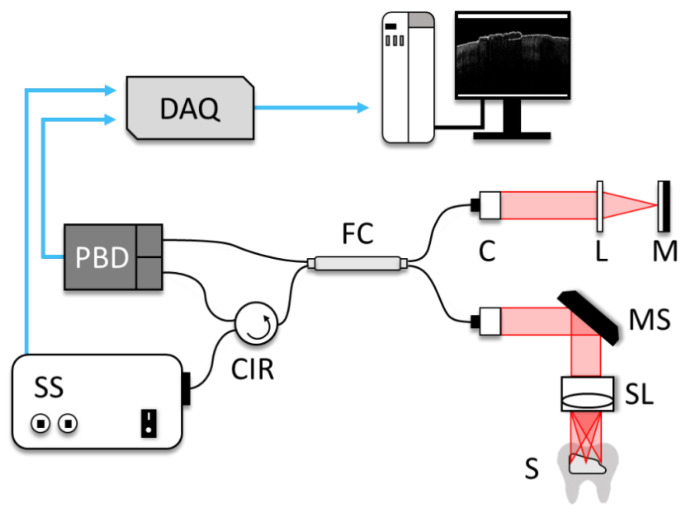
The schematic diagram of 1310 nm SS-OCT system. SS: swept-source, CIR: circulator, FC: fiber coupler, C: collimator, MS: MEMS scanner, L: lens, M: mirror, S: sample, SL: sample lens, BD: balanced detector.

During the assessment process, swept-source OCT system (SS-OCT, Thorlabs, Inc., Newton, NJ, USA, OCS1310V1) was used as the imaging tool to obtain all the multi-dimensional information including 2D cross-sections, 3D-volume renders, and in-front visualizations of the adhesive on the dental surface. The center wavelength of the laser was 1310 nm with a spectral bandwidth of 97 nm. The coherence length was 50 nm, and the axial scan rate 100 kHz. The axial and transverse resolutions of the system were 16 μm and 25 μm. The SS-OCT system schematic is shown in Figure 2. Sample field of view was 7 mm × 7 mm. The dimensions of the cross-sectional images were 712 × 713 pixels in the imaging modality. In order to obtain the thickness of the remaining adhesive, a custom algorithm was developed that extracted raw information from 2D-OCT cross-sectional images.

### 2.3. Description of the Customized Residual Adhesive Thickness and Area Detection Algorithm

**Figure 3:**
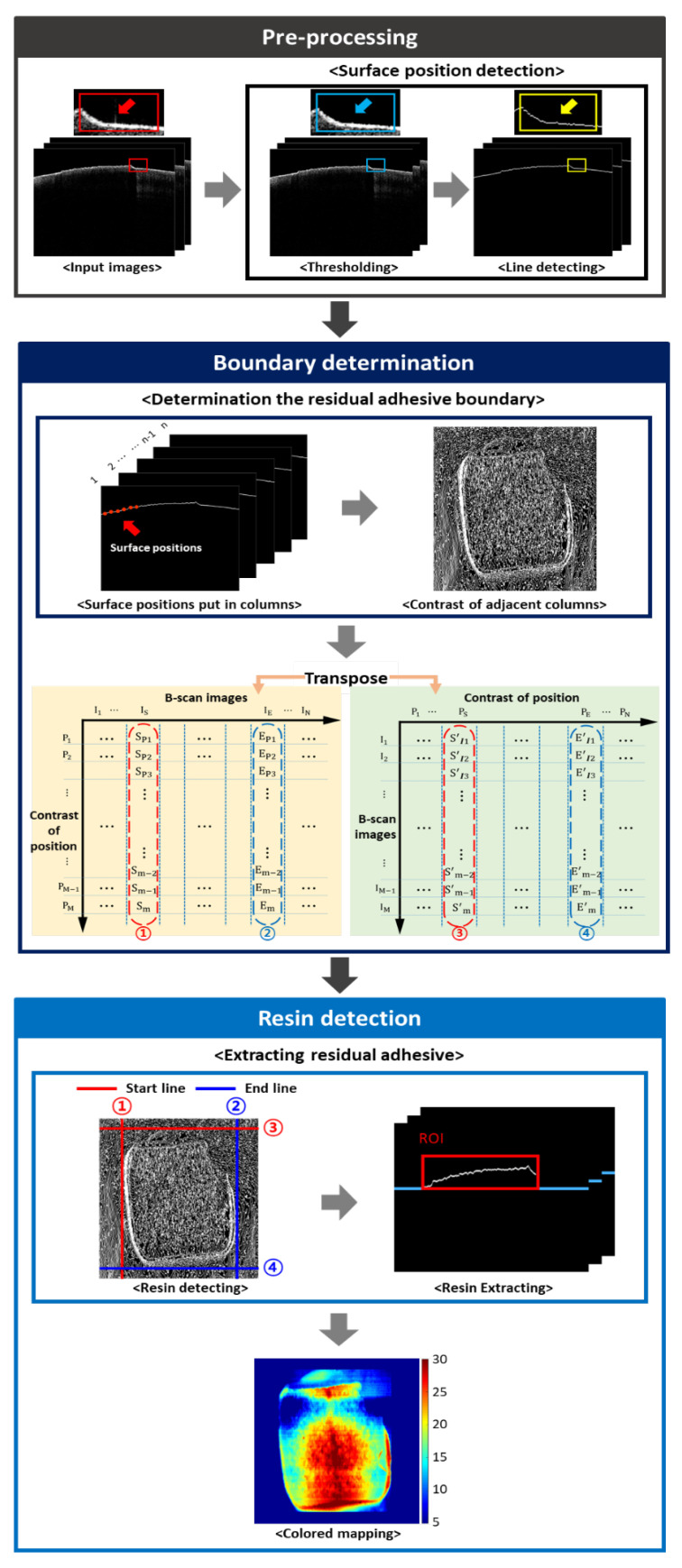
The flow chart of the residual adhesive detection procedure coded using MATLAB. The flow chart consists of three steps, such as pre-processing, boundary determination, and resin detection. In pre-processing, noise is removed and surface positions are detected. In boundary determination, the lines of boundary of residual adhesive are detected, while in resin detection, the residual adhesive from dental surface is extracted and quantitatively analyzes the area of residual adhesive.

To quantitatively analyze the thickness and area of residual adhesive on the tooth surface, a custom algorithm (coded in MATLAB, Natick, MA, USA) was developed. The custom algorithm shown in Figure 3 is divided into three steps, such as pre-processing, boundary determination and resin detection to capture the boundary, area, and thickness of the residual adhesive. Pre-processing is the initial step of the algorithm, which was applied to obtain original OCT images for making the line of tooth surface more clear to enhance the accuracy of the boundary determination process. Unnecessary noise generated by surface reflection at input images was removed through process, which eliminated each pixel with intensity lower than the surface value. When the intensity of pixels in OCT B-scan images were examined, it was indicated by a value between 0 and 255. At this time, the intensity of the first pixel on the surface of the tooth or the residual adhesive surface was checked and set as the threshold value. Due to the characteristics of the MATLAB (MathWorks, Natick, MA, USA) function, the pixel values were found from the first column of the array to the downward direction of the row, therefore, finding a value above the threshold value. The first pixel, with an intensity over the threshold value, was regarded as the position of the surface. Additionally, the pixels of the upward surface position pixel were 0 for removing the noise—‘thresholding process’. Thresholding process was applied from top to tooth surface and line detection was performed to obtain the surface pixel position of each line. Since noises were removed, the pixel position of tooth surface was determined as the position of drastic increment in intensity. According to the aforementioned method, each surface position of a single B-scan image was calculated and composed a single column of boundary matrix. Line detection was repeated until a 2D boundary matrix was obtained, yielding pixel positions for the whole volumetric image.

Next, boundary determination was performed to identify the residual adhesive boundary by utilizing the 2D boundary matrix obtained at pre-processing. The surface positions of B-scan images were stored in one matrix, which was used to find the boundaries of residual adhesive. Each column in the matrix represented the surface position of a single B-scan image, which took the contrast in these columns to obtain the boundary of the residual adhesive. By comparing adjacent columns, the algorithm checked the difference in surface height and found points that show a higher contrast in the tooth surface. Before applying the boundary determination process, the specific value of pixel intensity variance was pre-determined to distinguish the start and end position of residual adhesive. To find the beginning and end of the residual adhesive, the algorithm examines the contrast of all the columns, determining the first and last columns with contrast larger than the specific value as the boundary. These lines were consistent with the left ($I_{S}$) and right ($I_{E}$) edges of residual adhesive, which were marked as ① and ② shown in the left matrix of boundary determination (yellow background color). In addition to determining the top and bottom boundary of residual adhesive, an identical process of comparing position differences was performed for the transposed boundary matrix shown in the right matrix of boundary determination (green background color). As a result of boundary detection, obtained top ($P_{S}$) and bottom ($P_{E}$) boundaries were marked as ③ and ④.

In the concluding step of the custom algorithm, resin detection was applied to extract residual adhesive and to analyze thickness and area. To specify the residual adhesive, a resin detecting process used the edges of residual adhesive, which were found on the previous step. A region of interest (i.e., ROI) of rectangular shape was drawn from the vertices that met the boundaries of a single residual adhesive. Except for the interior of the rectangle, the outer part was classified as tooth surface and removed from the matrix. Then, residual adhesive thickness was obtained through the resin extracting process, which removed the unnecessary parts based on the lowest height of the ROI. A color mapping process was performed for the ROI to illustrate a correlation between color and thickness. The “jet” color scale of MATLAB was used on the colored in-front image. Thus, the colored in-front images emphasize the thickness information of residual adhesive—the thick point was red and the thin point was navy, as shown in a color bar. Since the residual adhesive thickness was different for each sample, the range of color scale was automatically regulated according to the obtained thickness information. To obtain the area of residual adhesive, we measured the area of a single pixel and multiplied this by the number of colored pixels.

## 3. Results

### 3.1. In-Front Visualizations of Residual Adhesive

**Figure 4:**
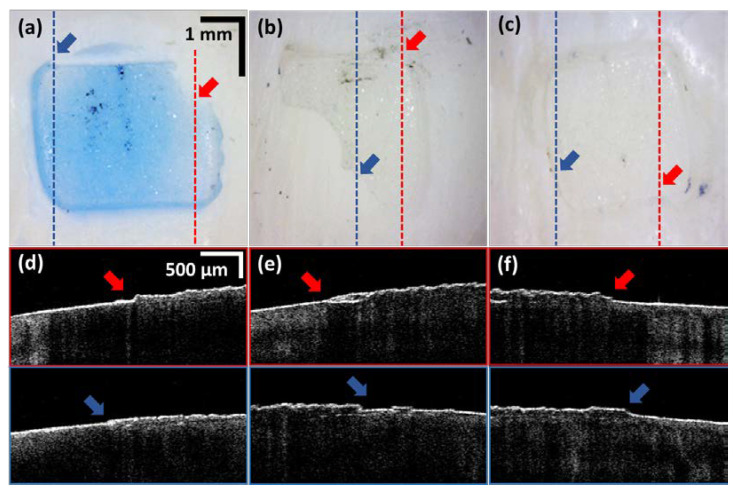
The images of the optical microscope of the samples and the cross-section images taken with OCT: (**a**–**c**) are images of optical microscope, and (**d**–**f**) are OCT cross-sectional images acquired from the regions marked with red and blue dash lines. The regions of thin residual adhesive, which are hard to be identified with the optical microscope, were clearly distinguished from OCT visualizations.

Figure 4 shows the optical microscopic images (acquired using a Dino camera, AM413T, Dino-Lite) and corresponding cross-sectional images of samples A, B, and C. A blue color adhesive was used in sample A, where the boundary between remaining adhesive and tooth can be distinguished to some extent through the naked eye. However, it was difficult to estimate the thickness of the residual adhesive. Samples B and C had the transparent adhesives commonly used in orthodontics. The boundaries of Figure 4a,c were barely distinguishable due to the transparency of the adhesive. The areas indicated by the blue and red dash lines with arrows on the images in Figure 4 represent the locations and boundaries of residual adhesive, which are hard to distinguish visually. OCT images corresponding to these particular locations illustrate the boundary between tooth and the residual adhesive.

### 3.2. Residual Adhesive Boundary Detection Algorithm-Based Quatitative Assessments

**Figure 5:**
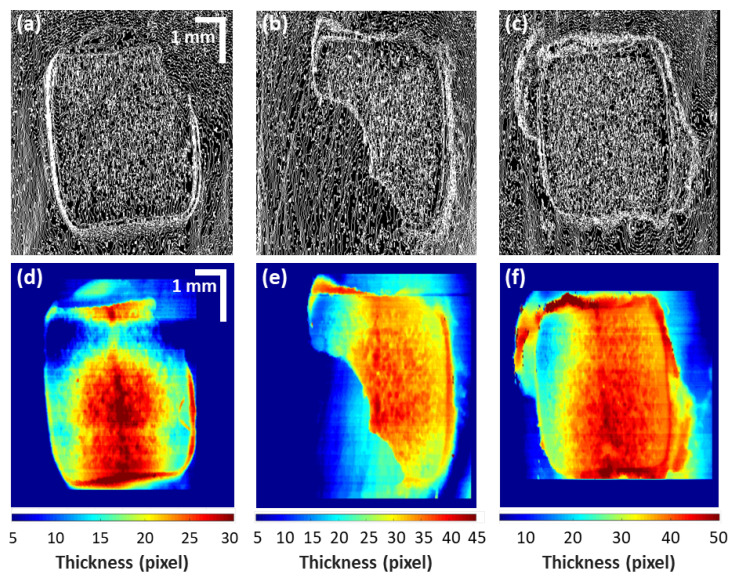
These are images obtained through custom algorithm: (**a**–**c**) are in-front images that compare the height differences on the surface of the OCT image for each sample; (**d**–**f**) are images that appear at the end of the algorithm, indicating only the areas judged to be residual adhesives, and expressing information about the thickness of the remaining adhesives in color.

Figure 5 illustrates the images obtained by storing the positions of the surface for each cross-sectional image of samples using the custom algorithm: surface in-front images of A, B, and C were compared to their respective outputs. The surface height of each cross-sectional image was stored as one column, and the stored columns were arranged to create in-front images Figure 5a–c. The subset images (Figure 5a–c) were further processed using the custom algorithm, to distinguish the contrast between adjacent lines. Due to the difference in intensity, distinguishable boundary region between tooth and residual adhesive can be clearly identified qualitatively and quantitatively from the acquired qualitative results. The interface between tooth and residual adhesive can be identified. When cross-sectional images of samples were inserted in the residual adhesive detection algorithm, the surface height of cross-sectional images is compared to determine the existence of residual adhesive. When cross-sectional images of samples were inserted in the residual adhesive detection algorithm, the surface height of cross-sectional images is compared to determine the existence of residual adhesive. If the amount of change in the surface height of the cross-sectional images exceeds a specific value, the existence of residual adhesive was assumed between the last exceeding positions.

Next, Figure 5d–f are the color-scaled images of sample A, B, and C, respectively. Colored images were generated according to the thickness of residual adhesive. These images visually represent the thickness of residual adhesive. To specify the residual adhesive, resin boundaries were used to sort the residual adhesive area and the other parts. A region of interest (i.e., ROI) of rectangular shape was drawn from the vertices that met the boundaries of a single residual adhesive. Except for the interior of the rectangle, the outer part is classified as a tooth surface and removed from the matrix, leaving only the residual adhesive inside the ROI. Then, thickness was calculated based on the height of the lower position, the beginning, and end height of the remaining adhesive. In order to leave only the thickness of the residual adhesive, the thickness value of the residual adhesive is corrected by subtracting the average value of the height change on the tooth surface. To intuitionally convey the thickness of residual adhesive to the observer in the next step, the regions with residual adhesive were colored according to the residual adhesive thickness. The color scale method was used to identify the maximum height of the residual adhesive and gradually set the color to change downward. This method provided intuitive thickness without the need for numerical values, allowing the actual observer to quickly determine the amount of residual adhesive for future treatment. Since the height of the residual adhesive is different, the scale also changed according to the residual adhesive height.

### 3.3. A Comparison between Optical Microscope and OCT Visualization

**Figure 6:**
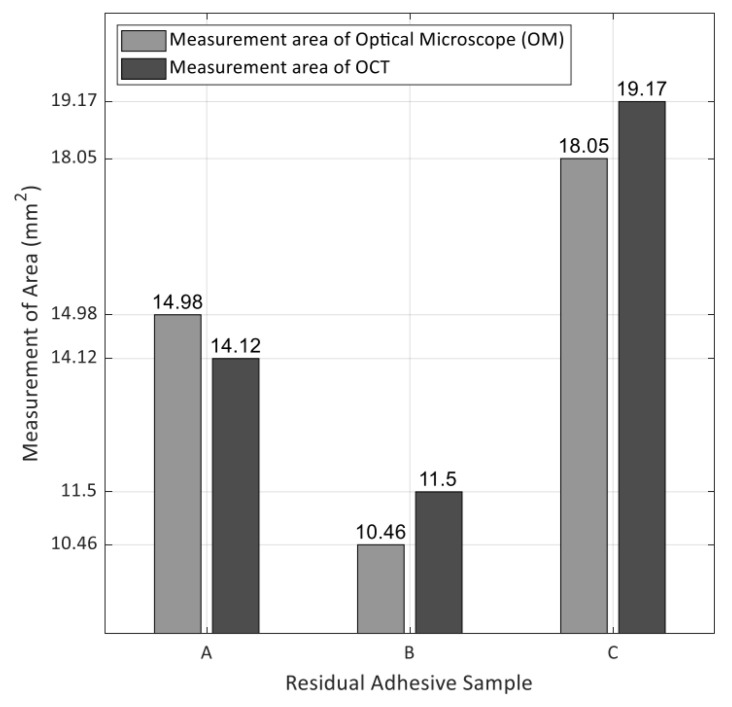
The bar graph shows the difference of residual adhesive between optical microscope and OCT image areas. The left bar of each sample represents the area of optical microscope measurement. The right bar of each sample represents the area of the OCT image. The grid of graph shows the difference in the area of each sample.

The optical microscope’s measurement area, and the residual adhesive area obtained by OCT images were compared to assess the accuracy of the algorithm’s residual adhesive detection capability. Optical microscope measurement area was obtained from the real sample image. In the algorithm, residual adhesive area was obtained from the OCT image by multiplying the one-pixel area for the number of colored pixels. For each sample, the measurement area of the optical microscope and the measurement area obtained by the algorithm were compared (Figure 6). A grid represented the difference in the area on the graph. The residual adhesive area obtained from the OCT image does not differ significantly from the optical microscope measurement area and the statistical analysis of the difference is shown in Figure 6 and Table 1. In sample A, OCT’s residual adhesive area was 0.862 mm^2^ smaller than the optical microscope measurement of residual adhesive (about 6% smaller). In sample B, the area of residual adhesive using OCT was 1.04 mm^2^ larger than the optical microscope measurement of residual adhesive (approximately 10% larger). The residual adhesive area of the OCT in sample C was 1.123 mm^2^ larger than the optical microscope measurement of residual adhesive (around 6% larger). Table 1 shows the exact number for each sample.

Therefore, the results reveal that the detection algorithm was quite accurate in detecting residual adhesive on the tooth surface and quantifying its amount using OCT. Unlike traditional qualitative and non-precise methodologies, this method provides accurate and rapid quantitative data and visual information. This method can be a starting point to the creation of new residual adhesive units.

## 4. Discussion

Regarding imaging tools, CT, micro-CT, and CBCT, were conventionally used in dentistry. Cosmin et al. [35] reported the analysis of the micro-CT imaging for orthodontic adhesive, with a comprehensive comparison between TD-OCT and micro-CT images, quantitatively analyzing the thickness of adhesive between teeth and bracket. Due to the lack of resolution of the utilized OCT, identifying the defects on the entire orthodontic attachments was challenging. However, the employed micro-CT images distinguished enamel-adhesive-bracket interface and enabled quantitative analysis. Through the result of micro-CT, thickness and width of adhesive were quantitatively analyzed, which resulted in a higher measurement of around 35%. In the current demonstration, quantitative analysis was conducted by imaging adhesive more accurately using an OCT engine with higher resolution and imaging speed in comparison with micro-CT. Furthermore, the errors of the quantifications were reduced by using the custom algorithm for residual adhesive. The result showed a difference of less than 10% from the area of the residual adhesive measured by microscope, and the color-scaled images intuitively revealed the thickness information of residual adhesive. Using the improved OCT system and the custom algorithm were the driving force behind the more accurate and quantitative analysis of residual adhesive. Due to the biological nature of the tooth specimen, a slight error was identified in each specimen during the analysis of area and thickness of the residual adhesive. Extensive technical modifications of the algorithm will be further performed secondarily to overcome these drawbacks in the succeeding attempt.

## 5. Conclusions

In this study, OCT-based quantitative algorithm was developed for the detection of residual adhesive on dental surface. In dentistry and orthodontics, CT has been used as the conventional imaging tool for detecting teeth structure and shape of malocclusion. Although CT has benefits in imaging, drawbacks, such as low resolution, and exposure to ionizing radiation are the main challenges. A microscope was alternatively used to find the decayed tooth or residual adhesive on dental surface, which has a limitation of examining sub-surface layers non-invasively. The application of OCT has succeeded in overcoming all the above-mentioned drawbacks by obtaining sub-surface information of specimens with a higher resolution than gold standards. Furthermore, developed custom algorithm enables the rapid identification of residual adhesive with in-front visualizations, and color-scaled images emphasize residual adhesive thickness information. The results of the proposed method are not limited to quantifications, but also acquire immediate feedback during the treatment progress with the colored in-front visualizations. These benefits of OCT and the developed custom algorithm have the potential of being used in clinics such as orthodontics and decayed teeth. In addition, the results sufficiently confirmed the potential of the developed method over gold standards in quantifying residual adhesives providing intuitive information, which is also expected to produce precise units over the existing ARI scores.

## Figures and Tables

**Table 1:** The actual area of the residual adhesive and the area of the residual adhesive obtained by the OCT image. Difference and ratio between the two areas.

Sample	Measurement Area of Optical Microscope (mm^2^)	Measurement Area of OCT (mm^2^)	Difference/Ratio
A	14.978	14.116	0.862/−6%
B	10.459	11.499	−1.04/+10%
C	18.051	19.174	−1.123/+6%

## Data Availability

The data presented in this study are available on request from the corresponding author.
